# The *Ntan1* gene is expressed in perineural glia and neurons of adult *Drosophila*

**DOI:** 10.1038/s41598-022-18999-8

**Published:** 2022-08-30

**Authors:** Ana Castañeda-Sampedro, Laura Calvin-Cejudo, Fernando Martin, Carolina Gomez-Diaz, Esther Alcorta

**Affiliations:** 1 Facultad de Medicina y Ciencias de la Salud, Departamento de Biología Funcional (Área de Genética), Universidad de Oviedo, c/Julián Clavería S/N, 33006 Oviedo, Asturias Spain; 2 Instituto de Neurociencias del Principado de Asturias (INEUROPA), Facultad de Medicina y Ciencias de la Salud, Universidad de Oviedo, Oviedo, Asturias Spain

**Keywords:** Cell biology, Genetics, Neuroscience

## Abstract

The *Drosophila Ntan1* gene encodes an N-terminal asparagine amidohydrolase that we show is highly conserved throughout evolution. Protein isoforms share more than 72% of similarity with their human counterparts. At the cellular level, this gene regulates the type of glial cell growth in *Drosophila* larvae by its different expression levels. The *Drosophila Ntan1* gene has 4 transcripts that encode 2 protein isoforms. Here we describe that although this gene is expressed at all developmental stages and adult organs tested (eye, antennae and brain) there are some transcript-dependent specificities. Therefore, both quantitative and qualitative cues could account for gene function. However, widespread developmental stage and organ-dependent expression could be masking cell-specific constraints that can be explored in *Drosophila* by using Gal4 drivers. We report a new genetic driver within this gene, *Mz317-Gal4*, that recapitulates the *Ntan1* gene expression pattern in adults. It shows specific expression for perineural glia in the olfactory organs but mixed expression with some neurons in the adult brain. Memory and social behavior disturbances in mice and cancer and schizophrenia in humans have been linked to the *Ntan1* gene. Therefore, these new tools in *Drosophila* may contribute to our understanding of the cellular basis of these alterations.

## Introduction

The *Ntan1* gene encodes the *Drosophila* N-terminal asparagine amidohydrolase homolog (NTAN1). It regulates endoreplication rates through the N-end rule pathway^1^. It has been proposed that in *Drosophila*, similarly to mammalian NTAN1^2^, this enzyme recognizes proteins with an N-terminal asparagine (Asn) and converts it to aspartate (Asp)^1^. After the action of other enzymes, such as ATE1, this site becomes then a potential substrate for ubiquitination and proteasomal degradation^1,3,4^.

At the cellular level, development of an organism consist on a myriad number of events that lead to the formation of an adult individual. Among these events, genetic regulation of cell number and size, controlled by growth and division^5^, is of extreme importance. In the nervous system, developmental processes need to strictly control and regulate glial and neuronal cell growth in order to achieve a functional organism.

In *Drosophila*, the surface of the entire nervous system is covered by two thin glial layers: the perineural glia (PNG) and the subperineural glia (SPG)^6^. Both types of glial cells are involved in the formation of the blood–brain barrier^7,8^. During larval growth, while the PNG grows exponentially in number by extensive cell division^9,10^ the SPG retains its small number and massively increases the size of its cells individually by both endoreplication and endomitosis becoming then polyploid^11,12^. It has been shown that in the blood–brain barrier of *Drosophila* the endoreplication of SPG cells is regulated by the *Ntan1* gene^1^, previously named *Öbek*.

In fact, in *Drosophila* larvae, glial cells present asparagine amidohydrolase activity of *Ntan1*^1^. The mechanism proposed in these cells for regulating their growth involves different expression levels of the *Ntan1* gene. During larval development, in SPG cells high expression levels of *Ntan1* limit cell cycle and replication rates controlling ploidy and number of nuclei^1^. On the contrary, PNG cells express moderate levels of *Ntan1*, allowing then efficient PNG proliferation^1^.

Although this gene has been thoroughly studied in larvae, a detailed description of gene expression that pays attention to its different transcripts both during development and in the adult phase is still lacking. In this article, we tackle this question by analyzing mRNA expression of the different transcripts of the *Ntan1* gene during different developmental stages and several adult organs such as the brain, eyes and antennae as representatives of central or peripheral nervous systems. Moreover, we show tissue-specific expression of the *Ntan1* gene in adults by using available Gal4 lines.

In the peripheral nervous system of *Drosophila,* such as that involved in olfactory reception, antennal glial cells begin to originate about 16 h after puparium formation (APF) and are closely associated with the development of sensory axons^13–15^. In the adult antennae, there are mainly two different types of glia, GH146-type and Mz317-type glia. GH146 glial cells correspond to ~ 30% of the glial antennal cells^15^. This glia originates in the brain and migrates to the third antennal segment where it ensheathes the axons of the ORNs which are projected from the antenna to the brain^15^. Mz317 glial cells corresponds to ~ 70% of antennal glial cells. They constitute a peripheral layer to GH146 glia and seem to surround the somas of olfactory sensory neurons^15^. Although Mz317::GFP is considered a marker of perineural and cell body glia labelling most of peripheral glia^16^, nothing is known about the molecular basis of this type of glia. In this report, we demonstrate that the Mz317 driver recapitulates the expression of the *Ntan1* gene as well as the other Gal4 drivers located within this gene.

## Results

### *Ntan1* gene is expressed in all developmental stages from embryo to adult *Drosophila*

**Figure 1:**
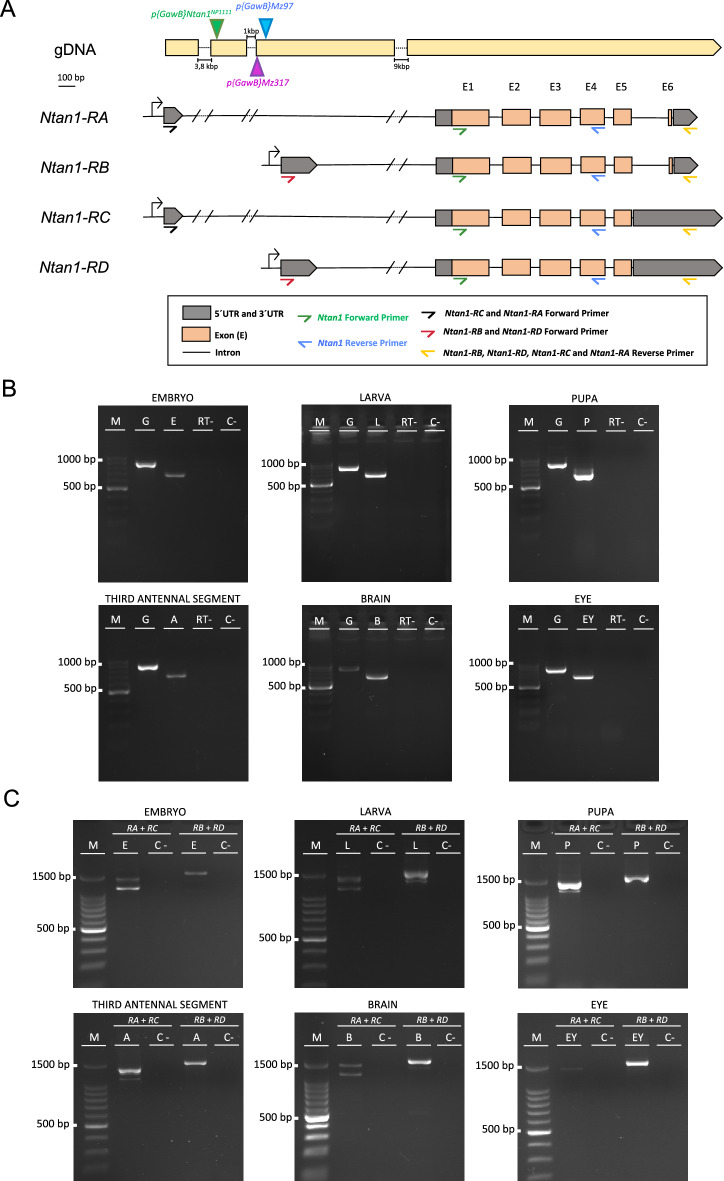
Gene expression analysis of the *Ntan1* gene in *Drosophila melanogaster*. (**A**) Schematic representation of the insertion sites of the Gal4 elements of *Mz317-Gal4*; *Mz97-Gal4,UAS-Stinger* and *Ntan1*^*NP1111*^*-Gal4* fly lines in the *Ntan1* gene. The *P{GawB}Mz317* insertion and the *P{GawB}Mz97* are located ∼ 150 bp and ∼ 100 bp upstream of the second transcriptional start site within the first intron, respectively. The *P{GawB}Ntan1*^*NP1111*^ insertion is located ∼ 1300 bp upstream of the second transcriptional start site within the first intron. 5′ UTR and 3′ UTR regions, exons and introns of each transcript and the location of the different primers used in RT-PCR are shown. (**B**) Gene expression of the *Ntan1* gene by RT-PCR in different developmental stages and adult organs of *wildtype* flies (*Canton-S*). (**C**) Gene expression of *Ntan1* transcripts by RT-PCR in different developmental stages and adult organs of *wildtype* flies (*Canton-S*). *M:* Molecular marker, *G:* Genomic DNA, *E*: Embryonic cDNA, *L:* Larval  cDNA, *P:* Pupal cDNA, *A:* Third antennal segment cDNA, *B: *Brain cDNA, *EY: *Eye cDNA, *RT−:* RT-PCR without retrotranscriptase, *C−:* Negative control.

Although it was previously shown that NTAN1 was strongly expressed in embryo and larvae of *Drosophila* in a subset of glial cells in both the PNS and the CNS along with a weakly expression in other tissues^1^, little is known about the *Ntan1* gene expression in other stages and adult organs. In order to test the native expression of the *Ntan1* gene in different pre-adult developmental stages (embryo, larva and pupa) and some adult organs (third antennal segments, brain and eyes) of *Drosophila melanogaster* we performed RT-PCR. As described in the material and methods section, primers were designed in order to amplify different size products from gDNA and cDNA (Fig. 1A, Table 4). The expected size for the cDNA of the *Ntan1* gene is 685 base pairs (bp), while for the genomic DNA is 901 bp. All products obtained in the *wildtype* genotype were of the expected sizes (Fig. 1B). These results confirm the expression of the *Ntan1* gene in all the developmental stages and adult organs studied.

### Gene expression of *Ntan1* transcripts at different developmental stages and adult organs

According to Flybase, the *Ntan1* gene has 4 transcripts: *Ntan1-RA*, *Ntan1-RB*, *Ntan1-RC* and *Ntan1-RD*, identical in their coding sequence two-by-two (*Ntan1-RA* and *Ntan1-RB*; *Ntan1-RC* and *Ntan1-RD*), but differentiable by their 5' and 3' UTR (*UnTranslated Region*) regions (Fig. 1A). The specific gene expression of each transcript of the *Ntan1* gene was analysed in the same developmental stages and organs previously mentioned. Thus being able to determine if there is one or several transcripts specific to a particular stage or organ tested. Amplification of genomic DNA was used as a PCR positive control. The expected cDNA sizes of *Ntan1-RA*, *Ntan1-RB*, *Ntan1-RC* and *Ntan1-RD* transcripts are 1136 bp, 1336 bp, 1405 bp and 1601 bp, respectively (Fig. 1C, Table 1). Table 1 summarizes the expression data for all transcripts in the different samples studied. *Ntan1-RA* transcript is expressed in embryo, larva, pupa, brain and third antennal segments but not in the adult eye whereas *Ntan1-RB* transcript is only expressed in larva and brain. Finally, both *Ntan1-RC* and *Ntan1-RD* transcripts are expressed in all developmental stages and organs studied (Table 1, Fig. 1C, Fig. S4).

**Table 1:** Summary of the results obtained in the gene expression analysis by RT-PCR of the *Ntan1* transcripts in different developmental stages and adult organs of *wildtype* genotype *Canton-S*.

Transcript	Embryo	Larva	Pupa	Adult		
				Brain	Eye	Third antennal segment
*Ntan1-RA*	+	+	+	+	−	+
*Ntan1-RB*	−	+	−	+	−	−
*Ntan1-RC*	+	+	+	+	+	+
*Ntan1-RD*	+	+	+	+	+	+

Negative symbol (−) indicates total absence of gene expression and positive symbol (+) indicates gene expression of the *Ntan1* transcripts.

### The *Ntan1* gene is highly conserved throughout evolution

**Figure 2:**
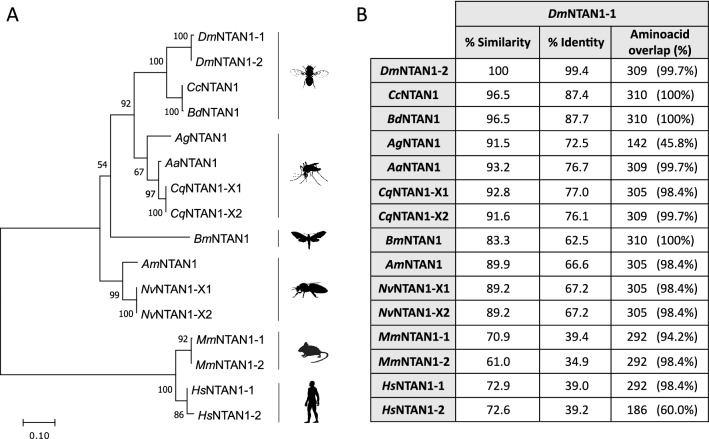
Comparative analysis of the aminoacid sequences of the NTAN1 isoforms of various insect and non-insect species. (**A**) Neighbor Joining tree of insects and other mammalian species NTAN1 orthologs. *Dm* (*Drosophila melanogaster*), *Cc* (*Ceratitis capitata*), *Bd* (*Bactrocera dorsalis*) *Ag* (*Anopheles gambiae*), *Aa* (*Aedes aegypti*), *Cq* (*Culex quinquefasciatus*), *Bm* (*Bombyx mori*), *Am* (*Apis mellifera*), *Nv* (*Nasonia vitripennis*), *Mm* (*Mus musculus*) and *Hm* (*Homo sapiens*). The tree was built with MEGA11. Bootstrap support (1000 replicates) is indicated. The scale bar units show aminoacid substitutions per site. (**B**) Comparison of sequence similarity and identity between the *Dm*NTAN1-1 isoform and the other isoforms of the different species studied with the LALIGN/PLALIGN software.

Although in *Drosophila* the *Ntan1* gene has four transcripts that differ mainly in their UTR regions, they are translated into two different isoforms. As N-terminal asparagine amidohydrolases have been found in other species, we performed a comparative analysis of the aminoacid sequences of the NTAN1 proteins isoforms of various insects belonging to the Diptera, Hymenoptera and Lepidoptera Orders as well as their corresponding mouse and human orthologs. We infered their evolutionary relationships by constructing a phylogenetic tree that clearly illustrates the evolutionary conservation of the products of the *Ntan1* gene (Fig. 2A). Although the percentage of identity between the *Drosophila melanogaster* NTAN1-1 isoform and the rest of ortholog isoforms from the species tested ranged from 34 to 87%, the percentage of similarity increases to the range between 61% to 96.5%. In fact, *Drosophila* and its human ortholog proteins share more than 72% of aminoacid similarity (Fig. 2B). In conclusion, we show that the *Ntan1* gene is highly conserved through evolution, not only among insects but also in mammals (Fig. 2). It is generally accepted that high conservation of genes along evolution indicates an essential functional role.

### *Mz317-Gal4* insert is located into the *Ntan1* gene and drives gene expression in adult brain and antennal glia

As it was previously shown that the *Ntan1* gene was involved in controlling the levels of ploidy in subperineural and perineural glial cells during larval development^1^ we wondered if this gene was also being expressed in adult perineural glia. The *Mz317-Gal4* line combined with a UAS-GFP reporter line drives the expression of GFP labeling the majority of peripheral glia including most antennal glia^15^ but the gene driving this especific mark was unknown. In order to determine the genomic location of the Gal4 insert of the *Mz317-Gal4* line we performed the Splinkerette technique^17^. This method allows to determine P-element insertion sites with unknown genomic location. By PCR amplification and posterior sequencing of the 3′ P-element insertion site (Figs. S1 and S2) we were able to conclude that the *Mz317-Gal4* line has its Gal4 element inserted into the *Ntan1* gene, specifically around 150 bp upstream the second 5′ UTR region.

It was known from previous studies that the *Mz317-Gal4* line drives gene expression in antennal perineural glia^15^ but in order to further investigate the cellular expression pattern of the *Mz317-Gal4* line in different cell types we analyzed GFP expression in the genotype *Mz317-Gal4;UAS-mCD8::GFP* both in antennal cryosections and brain dissections of *Drosophila melanogaster*. For this purpose, immunohistochemistry was performed with anti-GFP antibody on antennal cryosections and with anti-GFP and anti-nc82 antibodies on brain samples. The anti-nc82 antibody recognizes the neuropil-specific BRUCHPILOT protein, which allows us to visualize the general structure of the brain^18,19^. Also, to distinguish the specific cell types observed in the *Mz317-Gal;UAS-mCD8::GFP* line, we restricted GFP expression in either neurons or glia by adding gene expression restriction elements such as *elav-Gal80* and *repo-Gal80*, respectively. Genetic crosses were performed to obtain the following genotypes: *Mz317-Gal;UAS-mCD8::GFP* (GFP expression in all Mz317-type cells), *Mz317-Gal4/repo-Gal80;UAS-mCD8::GFP* (GFP expression absent in glia due to GAL80 expression driven by *repo*, a panglial marker), *Mz317-Gal4/elav-Gal80;UAS-mCD8::GFP* (GFP expression absent in neurons due to GAL80 expression driven by *elav*, a panneuronal marker). Immunostaining of the UAS-mCD8::GFP line by itself was used as a negative control.

**Figure 3:**
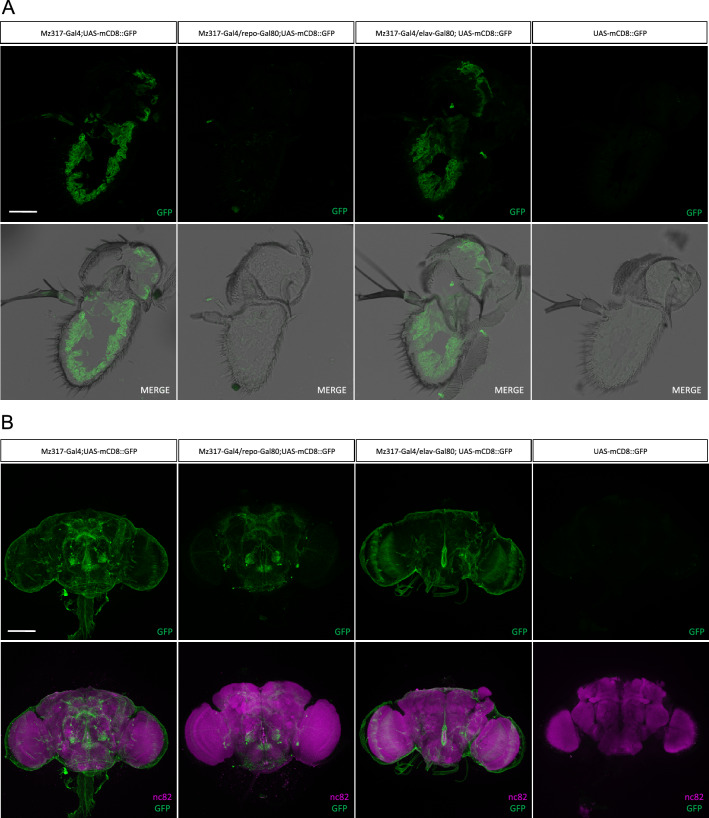
Analysis of GFP expression in the *Mz317-Gal4* line. (**A**) Immunohistochemistry on antennal cryosections (14 µm) with anti-GFP antibody (green) on the following genotypes: *Mz317-Gal4;UAS-mCD8::GFP*, *Mz317-Gal4/repo-Gal80;UAS-mCD8::GFP*, *Mz317-Gal4/elav-Gal80;UAS-mCD8::GFP* and *UAS-mCD8::GFP*. Scale bar represents 40 µm. (**B**) Immunohistochemistry on brain dissections with anti-GFP (green) and anti-nc82 (magenta) antibodies on *Mz317-Gal4;UAS-mCD8::GFP*, *Mz317-Gal4/repo-Gal80;UAS-mCD8::GFP*, *Mz317-Gal4/elav-Gal80;UAS-mCD8::GFP* and *UAS-mCD8::GFP*. Scale bar represents 100 µm.

In the antenna, the *Mz317-Gal4* line directs GFP expression exclusively in glial cells, as we did not observe any GFP expression in antennal neuronal cells (Fig. 3A). Furthermore, the antennal GFP expression pattern in *Mz317-Gal4/elav-Gal80;UAS-mCD8::GFP,* that restricts Ntan1-drived expression to non-neuronal cells, matches exactly the GFP expression pattern of the *Mz317-Gal;UAS-mCD8::GFP* individuals previously described^15^ (Fig. 3A). Antennal GFP expression is observed at the base of the sensilla, most likely surrounding the neuronal somas of most olfactory sensory neurons. Likewise, GFP expression is observed around the olfactory nerve. Thus, the expression pattern of the *Mz317-Gal4* line corresponds exclusively to perineural antennal glial cells.

As expected, GFP expression is clearly observed as a wrap around the whole brain structure (Fig. 3B). Additionally, we also observe staining in a selected number of non-glial cells in the brain (Fig. 3B). These cells correspond to neuronal cells as inferred from the restriction of GAL4 expression in *elav-Gal80* expressing cells that suppressed this staining. These neurons are present in limited numbers in the subesophageal ganglion (SNG) (15 ± 2 cells), the mushroom bodies (MB) area and the upper part of the protocerebrum (SP) (12 ± 2 cells), the antero-ventrolateral part (AVLP) and projecting neurons of the optic lobes (OL) (32 ± 4 cells), and to an area close to the antennal lobes (AL) (6 ± 0 cells) where olfactory interneurons are usually located.

In summary, the *Mz317-Gal4* line directs gene expression in the antenna exclusively in perineural glia and in the brain in perineural glial cells and in a limited number of neuronal cells.

### *Ntan1* gene as a marker for antennal and brain perineural glia

Once it was clear that the Gal4 insert of the *Mz317-Gal4* line was located into the *Ntan1* gene we wondered if there were other genetic driver lines with inserts in the *Ntan1* gene that showed a similar expression pattern. Two other lines have driver insertions in the *Ntan1* gene, *Mz97-Gal4* and *Ntan1*^*NP1111*^*-Gal4*. In both cases, the Gal4 elements are inserted in the first intron of the *Ntan1* gene (^1^ and https://flybase.org/ respectively). Note that the non-transcribed genomic DNA that is located in the middle of both 5’UTRs is considered the first intron according to the Ensembl database. While the *Mz97-Gal4* insert is located ~ 100 bp upstream the second transcriptional start site the insertion of the *Ntan1*^*NP1111*^*-Gal4* is located ~ 1300 bp upstream this region (Fig. 1A).

In order to compare the expression patterns of these Gal4 lines with *Mz317-Gal4* we performed immunohistochemistry with anti-GFP antibody in the antenna and anti-GFP and anti-nc82 in the brain in the following genotypes: *Mz317-Gal4;UAS-mCD8::GFP*, *Ntan1*^*NP1111*^*-Gal4;UAS-mCD8::GFP* and *Mz97-Gal4,UAS-Stinger;UAS-mCD8::GFP*. In the last genotype, cell nuclei will also be GFP-stained together with membrane-bound GFP (*mCD8*) due to the additional UAS-Stinger element that is also present in the original MZ97-Gal4 line.

**Figure 4:**
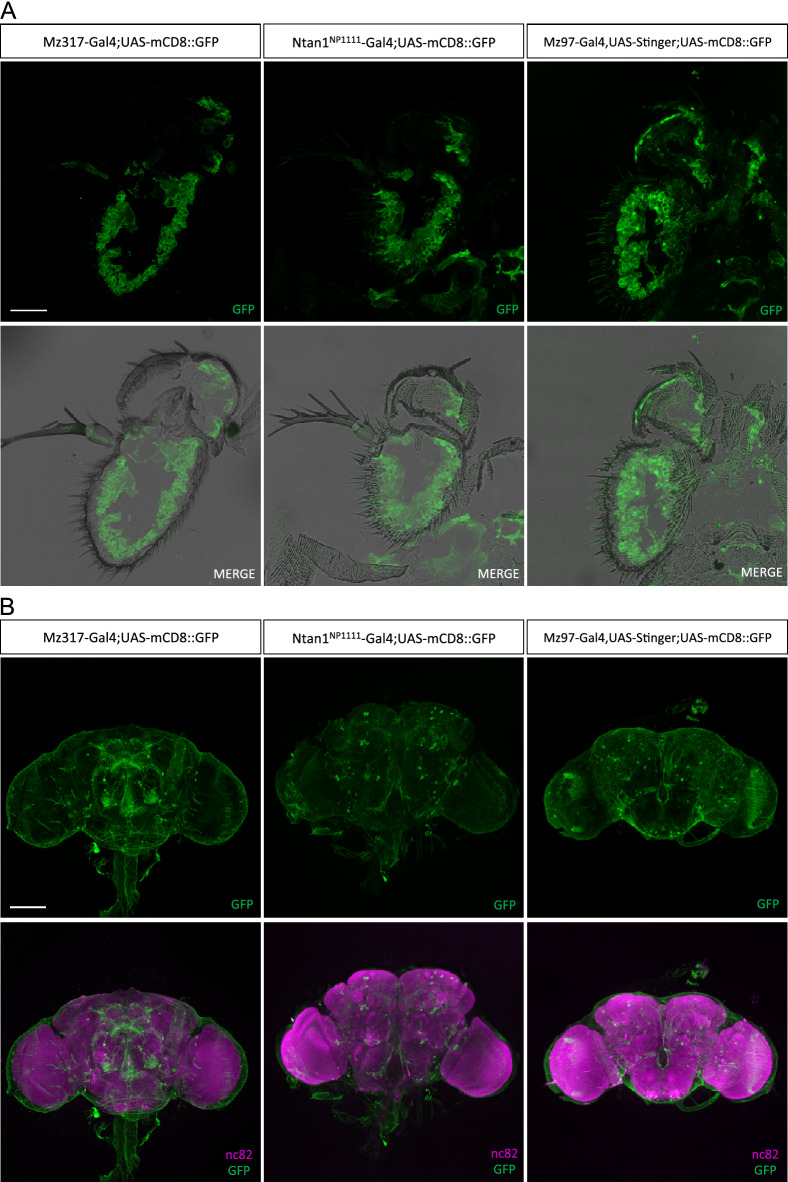
Analysis of GFP expression in different *Ntan1*-related Gal4 lines in *Drosophila melanogaster*. (**A**) Immunohistochemistry on antennal cryosections (14 µm) with anti-GFP antibody (green) on the following genotypes: *Mz317-Gal;UAS-mCD8::GFP*, *Mz97-Gal4,UAS-Stinger;UAS-mCD8::GFP* and *Ntan1*^*NP1111*^*-Gal4;UAS-mCD8::GFP*. Scale bar represents 40 µm. (**B**) Immunohistochemistry on brain dissections with anti-GFP (green) and anti-nc82 (magenta) antibodies on *Mz317-Gal;UAS-mCD8::GFP*, *Mz97-Gal4,UAS-Stinger;UAS-mCD8::GFP* and *Ntan1*^*NP1111*^*-Gal4;UAS-mCD8::GFP*. Scale bar represents 100 µm.

The antennal GFP expression observed was very similar in the three lines tested (Fig. 4A). Both in *Mz97-Gal4* and *Ntan1*^*NP1111*^*-Gal4* we observed glial GFP staining located in the base of the sensilla of the third antennal segment, most likely surrounding the neuronal somas of the olfactory sensory neurons as in *Mz317-Gal4* (Figs. 3A and 4A). In the brain, there are slight differences in GFP expression among the different fly lines (Fig. 4B). All brains appear to have a glial wrap around their structure. However, *Ntan1*^*NP1111*^*-Gal4;UAS-mCD8::GFP* and *Mz97-Gal4,UAS-Stinger;UAS-mCD8::GFP* brains show a lower number of neuronal cells in the superior protocerebrum area, the mushroom body area and in the vicinity of the antennal lobes compared to *Mz317-Gal4;UAS-mCD8::GFP* (Fig. 4B). In the case of the *Mz97-Gal4,UAS-Stinger;UAS-mCD8::GFP* line there seems to be a higher expression of GFP, however, this is due to *Stinger* expression which also marks the cell nuclei. Taken together, these results indicate that the *Ntan1* gene is mainly expressed in both antennal and brain perineural glia.

### Gene expression of *Ntan1* transcripts in different Gal4 lines

**Figure 5:**
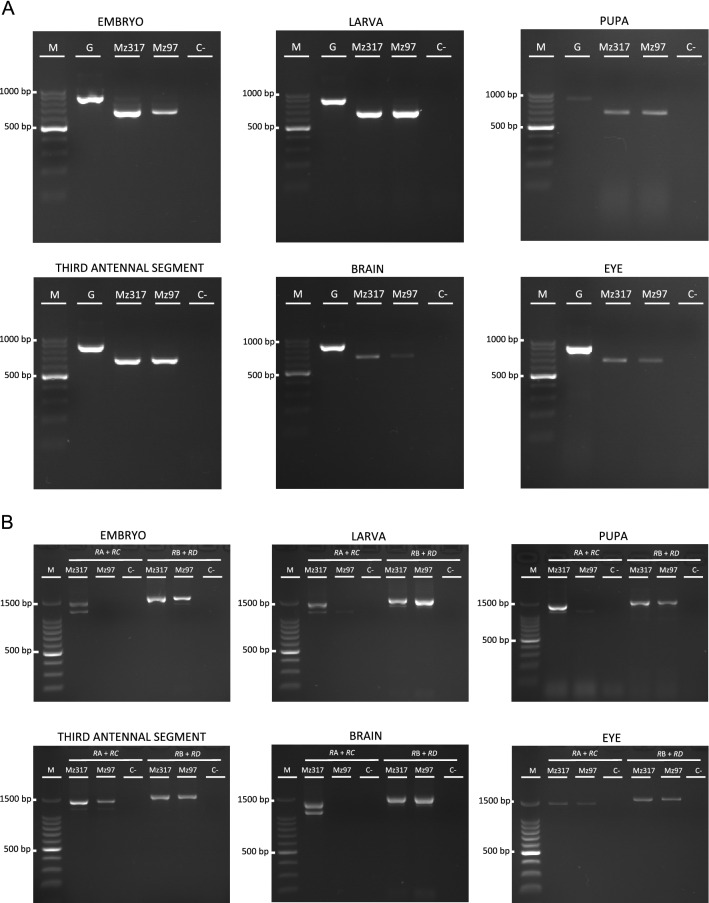
Gene expression analysis of *Ntan1* in *Mz97-Gal4* and *Mz317-Gal4* fly lines. Expression of the *Ntan1* gene (**A**) and *Ntan1* transcripts (**B**) by RT-PCR in *Mz97-Gal4* and *Mz317-Gal4* fly lines. *M:* Molecular marker, *G:* Genomic DNA of *wildtype* genotype, Mz317: *Mz317-Gal4 cDNA*, Mz97: *Mz97-Gal4,UAS-Stinger* cDNA and C−: Negative control.

After determining the developmental stages and organs in which the different transcripts of *Ntan1* were expressed (Fig. 1C, Table 1) we wondered if the slight differences observed in brain staining among the different driver lines tested could be due to qualitative differences in transcript expression in the fly lines tested. For this purpose, we studied the expression of *Ntan1* gene in different developmental stages and adult organs of *Mz317-Gal4* and *Mz97-Gal4,UAS-Stinger* lines. In addition, we have determined whether or not there are one or more transcripts specific to a particular stage or adult organ in each fly line. As the Gal4 element of *Mz97-Gal4,UAS-Stinger* and *Ntan1*^*NP1111*^*-Gal4* lines is inserted in the same area (first intron), the gene expression analysis of *Ntan1* gene has been carried out only in the *Mz97-Gal4,UAS-Stinger* line. As before, an RT-PCR experiment was performed to check the native expression of the *Ntan1* gene in *Mz317-Gal4* and *Mz97-Gal4,UAS-Stinger* lines. Genomic DNA amplification was used as a positive control. The expected size of cDNA of the *Ntan1* gene was 685 bp using the primers Ntan1-F GGTGTGCTGCTGCAGGATGACT and Ntan1-R CGTAGTTAAAGGGTCCAATGC. In both lines tested, the PCR products obtained had the expected sizes (Fig. 5A). These results confirm the expression of the *Ntan1* gene in the different stages and organs studied in *Mz317-Gal4* and *Mz97-Gal4,UAS-Stinger* lines. The expression of each transcript was also evaluated in both lines (Fig. 5B). Although in the *wildtype* genotype *Ntan1-RB* transcript is not expressed in embryo and pupa our results show that all transcripts are expressed in embryo, larva and pupa of *Mz317-Gal4* line (Table 2). Also, some differences are observed in relation to *Mz97-Gal4,UAS-Stinger* line and the *wildtype* genotype. Specifically, there is no expression of *Ntan1-RA* transcript in embryo of *Mz97-Gal4,UAS-Stinger* line and additionally, there is no expression of *Ntan1-RC* transcript in embryo, larva or pupa of *Mz97-Gal4,UAS-Stinger* line (Table 2). On the other hand, in adult *Drosophila*, *Ntan1-RA* transcript is expressed in the brain and third antennal segments of the *Mz317-Gal4* line, while it is only expressed in third antennal segments of *Mz97-Gal4,UAS-Stinger*. *Ntan1-RB* transcript is expressed only in the brain of both lines, whereas *Ntan1-RC* transcript is expressed in the brain, third antennal segments and eyes of the *Mz317-Gal4* line and third antennal segments and eyes of the *Mz97-Gal4,UAS-Stinger* line. Finally, *Ntan1-RD* transcript is expressed in all three organs studied of both lines similarly to the *wildtype* genotype (Table 3). To sum up, the main gene expression differences in adult *Drosophila* between the *wildtype* genotype and the Gal4 lines studied are seen in the expression of *Ntan1-RA* and *Ntan1-RC* transcript of *Mz97-Gal4,UAS-Stinger* in the brain (Table 3). We observed no expression of the transcript *Ntan1-RB* in the embryo and in the pupa of the *wildtype* genotype (Fig. 1C, Table 1), whereas there is expression of this transcript in the Gal4 lines studied (Fig. 5B, Table 2). We performed an additional PCR using the *G6PD* housekeeping gene for the embryo and brain of the *Mz97-Gal4* fly line, where we did not observe any expression of *Ntan1-RA* and *Ntan1-RC* (Fig. S3). With this data we can infer that even slight differences in the insertion sites of P(GawB) driver elements change gene expression of the *Ntan1* transcripts that might account for slight differences also in GFP expression drived by the lines tested.

**Table 2:** Summary of the results obtained in the gene expression analysis by RT-PCR of the *Ntan1* transcripts in different development stages (embryo, larva and pupa) in different genotypes.

Transcript	Embryo			Larva			Pupa		
	*wt*	*Mz317*	*Mz97*	*wt*	*Mz317*	*Mz97*	*wt*	*Mz317*	*Mz97*
*Ntan1-RA*	+	+	−	+	+	+	+	+	+
*Ntan1-RB*	−	+	+	+	+	+	−	+	+
*Ntan1-RC*	+	+	−	+	+	−	+	+	−
*Ntan1-RD*	+	+	+	+	+	+	+	+	+

The genotypes tested are: *Canton-S* (wt), *Mz317-Gal4* (Mz317) and *Mz97-Gal4,UAS-Stinger* (Mz97). Negative symbol (−) indicates total absence of gene expression and positive symbol (+) indicates gene expression of the *Ntan1* transcripts.

**Table 3:** Summary of the results obtained in the gene expression analysis by RT-PCR of the *Ntan1* transcripts in different adult organs in different genotypes.

Transcript	Adult								
	Brain			Eye			Third antennal segment		
	*wt*	*Mz317*	*Mz97*	*wt*	*Mz317*	*Mz97*	*wt*	*Mz317*	*Mz97*
*Ntan1-RA*	+	+	−	−	−	−	+	+	+
*Ntan1-RB*	+	+	+	−	−	−	−	−	−
*Ntan1-RC*	+	+	−	+	+	+	+	+	+
*Ntan1-RD*	+	+	+	+	+	+	+	+	+

The genotypes tested are: *Canton-S* (wt), *Mz317-Gal4* (Mz317) and *Mz97-Gal4,UAS-Stinger* (Mz97). Negative symbol (−) indicates total absence of gene expression and positive symbol (+) indicates gene expression of the *Ntan1* transcripts.

## Discussion

In this article, we offer a detailed gene expression study of the *Ntan1* gene, previously characterized as encoding an N-terminal asparagine amidase in *Drosophila*^1^ as well as in other species of invertebrates and vertebrates^2^. At the cellular level its role has been related to the control of cell cycle and replication rates controlling ploidy and number of nuclei in some cellular types, especially in glia. In this cell type moderate expression levels associate to perineural glial cells (PNG) while high expression levels appear in subperineural glial cells during larval development in *Drosophila*^1^. In summary, the quantitative expression of the gene rather than just its presence or absence appears to be at the basis of its function deduced from studies of the entire gene. However, the gene encodes several transcripts and the importance of qualitative differences in expression depending on the developmental stage and cell type cannot be excluded beforehand.

In this report we study the expression of the *Ntan1* gene and each of its 4 transcripts *− RA* to *− RD* during different developmental stages and in some adult organs to see if there is a specific expression pattern for any of them. It should be reminded that the differences in the sequence of the transcripts mainly affect their regulatory regions rather than the coding sequence that is identical between *− RA* and *− RB* (isoform 1) and between *-RC* and *-RD* (isoform 2), respectively. The results indicate that although there is expression of the *Ntan1* gene in all stages and organs tested there are some specificities in the transcript expression (Fig. 1B,C, Table 1). Looking at the different developmental stages (embryo, larva and pupa), *− RA*, *− RC* and *− RD* transcripts appear always, only the − RB transcript shows expression in the larva but does not express in embryo and pupa. In the adult stage the *Ntan1-RB* transcript is possibly specific to the brain, as it does not appear in neither the third antennal segment nor the eye. As for the other transcripts *Ntan1-RA*, *Ntan1-RC* and *Ntan1-RD* show generalized expression in the adult organs with the only exception of *− RA* that does not show expression in the eye. This means that the eye lacks isoform 1 as it expresses neither *− RA*, nor *− RB*. This is not unprecedented, as previous reports in *Drosophila* on other protein families such as G-proteins have shown specificities associated with eye-related tissues^20–22^.

However, it is important to highlight that although the coding sequence of the *Ntan1-RB* transcript is identical to the sequence of *Ntan1-RA* transcript (Fig. 1A) they differ in their regulatory regions, 5′ UTR and 3′ UTR. Thus, these differences in transcript expression might account for the different expression levels of the *Ntan1* gene that drive different cell growth mechanisms in PNG cells and the SPG cells^1^. In vertebrates a similar behavior has been reported for this gene, as variable expression of an approximately 1.4-kb transcript was reported in all 8 mouse tissues examined, with lowest expression in testis. However, a transcript of about 1.1 kb was prominently expressed in testis only^2^.

By attending to the two characteristics, transcript and isoform type, qualitative and quantitative determination seems to play an important role in gene function. In *Drosophila* there are some organs such as the compound eyes that express only one *Ntan1* isoform, while in the other cases analyzed both are expressed, but including either one or both transcripts. However, it should not be forgotten that both the different developmental stages studied and the adult organs include a variable number of cell types, so that the data obtained as a whole may be masking specificities at the cellular level. Some data in this direction was reported in mouse, where the *Ntan1* expression decreased following differentiation in C2C12 mouse myoblasts, but not in MEL mouse leukemia cells^2^.

Detailed studies at the cellular level are difficult to address in small organisms by RNA expression analysis when the gene studied shows widespread expression in different cell types and quantitative issues play an important role. In such cases, immunohistochemical analysis with the gene product does not usually give consistent results either. In *Drosophila*, thanks to the development of numerous genetic tools such as the Gal4/UAS system and its variants, this analysis can be done by selective labeling with reporter genes such as GFP whose expression is directed with drivers that recapitulate the expression pattern of the gene under study^23,24^. In many cases, this is obtained by insertions within the gene. These tools can even serve to follow gene expression in different cell types throughout development like in the G-TRACE method^25,26^, as well as to direct the generation of functional mutants restricted to some cell types. However, the validity and significance of the data obtained depends on the accuracy and reliability with which these drivers recreate the expression of the genes of interest.

In this sense, we have located a new driver within the *Ntan1* gene (*Mz317-Gal4* line) and we have studied the expression of the gene in this line compared to *wildtype* flies. Moreover, we have described the expression patterns in glia and neurons of other driver lines that had already been described by their insertion within this gene (*Mz97-Gal4* and *Ntan1*^*NP1111*^), in order to characterize how they recapitulate the expression of the original gene and can be used for further studies.

In this work we report for the first time that the *Mz317-Gal4* line^16^ has an insert within the *Ntan1* gene. According to Sen et al.^15^ Mz317::GFP is a marker for perineural glia that labels the majority of peripheral glia. We characterized the expression of *Ntan1* in the *Mz317-Gal4* line both at the molecular and cellular level. RT-PCR analysis of the adult organs in the *Mz317-Gal4* line recapitulates the same expression pattern that the *wildtype* individuals.

At the cellular level, immunohistochemistry in the third antennal segment and brain of *Mz317-Gal4;UAS-mCD8::GFP* individuals reveals the cell types marked in both structures. Although we detected specific expression of perineural glia in the third antennal segment, in agreement with what has been previously reported^15^, the labeling in the brain includes mostly glial cells and a reduced number of neurons.

We also compared *Ntan1* gene expression in *wildtype* and *Mz317-Gal4* individuals with that obtained with other drivers previously mapped to the *Ntan1* gene, both at the molecular and cellular levels. The transcript *Ntan1-RB* is not expressed neither in the embryo nor in the pupa of the *wildtype* genotype whereas there is expression of this transcript in the Gal4 lines studied. In adults, the expression pattern of the *Mz317-Gal4* line matches that of *wildtype* individuals while the *Mz97-Gal4,UAS-Stinger* line shows no expression of the *-RA* or *-RC* transcript in the brain. In a previous work, it was indicated that the *Mz97-Gal4* insertion might have led to a mutant *Ntan1* allele^1^. In homozygous *Mz97-Gal4* flies there is still expression of various transcripts of the *Ntan1* gene, although quantitative differences in gene expression might not be detected by the methodology used. Our data suggest that a mutant phenotype may be generated if it depends on the quantitative expression of either or both *Ntan1* isoforms in the brain.

At the cellular level, the expression patterns of the different Gal-4 lines mapped to the *Ntan1* gene are very similar. In the third antennal segment, expression is restricted to PNG glia, whereas in the brain staining shows as a glial wrap with the exception that *Ntan1*^*NP1111*^*-Gal4;UAS-mCD8::GFP* and *Mz97-Gal4,UAS-Stinger;UAS-mCD8::GFP* show a lower number of neurons than *Mz317-Gal4;UAS-mCD8::GFP*.

The differences in gene expression among the different lines, both at the molecular and cellular level, relate to the different positions of the Gal4 insertion that may lead to mutations in some cases. This indicates the importance of performing previous expression studies if we want to use Gal4 insertion lines as tools for the study of gene expression as well as to generate cell type-specific mutations. However, the difficulties in targeting a certain cell type can be solved in some cases with additional elements like the Gal80 inserts.

Summarizing, we consider that the *Ntan1* gene could be easily used as a marker and/or driver of just perineural glia, by restricting neuronal expression with a Gal80 insertion. Therefore, with this work we show that the Gal4 lines associated *to* the *Ntan1* gene in *Drosophila* will add new genetic tools for studying the regulation and function of glia.

Glia is essential in the development and homeostasis of the nervous system due to its interaction with neurons and its modulatory role in neuronal activation^27^. In mammals, glia can constitute between 33 and 66% of the brain mass^28,29^. However, in *Drosophila*, glia makes up approximately 10% of the cells in the nervous system^6^. The low percentage of glial cells relative to other species and the similarity of its nervous system to vertebrates^27,30,31^, make *Drosophila melanogaster* an excellent model organism to investigate the biological mechanisms behind glial regulation of the nervous system.

Finally, it has been shown that the *Ntan1* gene performs numerous functions in vertebrates and defects in the gene can have serious effects at the level of the organism. Thus, alterations in the *Ntan1* gene expression have been associated with different types of memory and social behavior^32,33^, sporadic childhood cancer^34^ and risk of schizophrenia^35^. Therefore, studies in *Drosophila* using all the new tools available achieve a greater projection in determining the cellular basis of these phenotypes, which is necessary for their possible reversion.

## Materials and methods

### *Drosophila* lines

Flies were maintained in a thermoregulated chamber at 25 ± 1 °C in 12 h light:12 h dark conditions. The following fly lines were used in this work: *Canton-S* (Bloomington *Drosophila* Stock Center, BDSC 64349), *Mz97-Gal4,UAS-Stinger* (BDSC 9488), *UAS-mCD8::GFP* (BDSC 5130), *Ntan1*^*NP1111*^*-Gal4* (Kyoto Stock Center, DGRC 103887), *Mz317-Gal4*^16^, *repo-Gal80* and *elav-Gal80* (kindy provided by Leiserson, W., Yale University, USA; Lee, T. Janelia Research Campus, USA; Casas-Tintó, S. Instituto Cajal CSIC, Madrid, Spain, respectively).

### Phylogenetic analysis

Aminoacid sequences of NTAN1 of *Drosophila melanogaster* (*Dm*), *Ceratitis capitata* (*Cc*), *Bactrocera dorsalis* (*Bd*), *Anopheles gambiae* (*Ag*), *Aedes aegypti* (*Aa*), *Culex quinquefasciatus* (*Cq*), *Bombyx mori* (*Bm*), *Apis mellifera* (*Am*), *Nasonia vitripennis* (*Nv*), *Mus musculus* (*Mm*) and *Homo sapiens* (*Hm*) were obtained from the National Center for Biotechnology Information (NCBI) database (*Dm*NTAN1-1: NP_611355.2; *Dm*NTAN1-2: NP_001163199.1; *Bd*NTAN1: XP_011208810.1; *Ag*NTAN1: XP_001230643.2; *Aa*NTAN1: XP_021711016.1; *Cq*NTAN1-X1: XP_038110105.1; *Cq*NTAN1-X2: XP_038110106.1; *Bm*NTAN1: XP_037869646.1; *Am*NTAN1: XP_016769476.1; *Nv*NTAN1-X1: XP_032456886.1; *Nv*NTAN1-X2: XP_032456890.1; *Mm*NTAN1-1: NP_035076.1; *Mm*NTAN1-2: NP_001333035.1; *Hs*NTAN1-1: NP_775745.1; *Hs*NTAN1-2: NP_001257695.1). All sequences were aligned with MUSCLE using default parameters. The alignment was used to build a Neighbor Joining tree with evolucionary distances computed using the Jones, Taylor and Thorton matrix-based method^36^, 1000 bootstrap replicates and gaps handled with pairwise deletion. Phylogenetic analyses were conducted in MEGA11^37^.

### Immunostaining

Immunofluorescence on antennal cryosections were performed as described in^38^. Briefly, 14 µm antennal cryosections were fixed in 4% paraformaldehyde (Electron Microscopy Sciences, Hatfield, Pennsylvania) in Phosphate Buffered Saline (PBS) for 7 min. Antennal cryosections were washed two times with PBS and incubated with PBST (PBS with 0.1% Triton X-100, Sigma-Aldrich, Saint Louis, USA) for 30 min. Then, the samples were incubated in PTS (5% heat inactivated normal goat serum (Biowest, Nuaillé, France) in PBST) for 30 min. After that, samples were incubated overnight at 4 °C in dark with anti-GFP rabbit primary antibody (1:5000; Invitrogen, Eugene, USA) diluted in PTS. Samples were washed 10 min for three times in PBST and incubated in PTS for 30 min. Subsequently, cryosections were incubated with Alexa 488 anti-rabbit secondary antibody (1:1000; Invitrogen, Eugene, USA) diluted in PTS for 2 h at room temperature in dark. Finally, after several washes with PBST, samples were mounted using Vectashield mounting medium (Vector Laboratories, Burlingame, USA).

To detect GFP expression in the brain, immunohistochemical experiments were carried out on whole brain dissections previously fixed in 4% paraformaldehyde (Electron Microscopy Sciences, Hatfield, Pennsylvania) in PBS with 0.2% Triton X-100 (Sigma-Aldrich, Saint Louis, USA) for 3 h at 4 °C following the protocol described in^39^. After several washes in PBST 0.2% (PBS with 0.2% Triton X-100, (Sigma-Aldrich, Saint Louis, USA), brains were dissected in blocking solution (5% heat inactivated normal goat serum (Biowest, Nuaillé, France) in PBST 0.2%). Dissected brains were incubated with primary antibodies diluted in blocking solution: anti-GFP rabbit (1:2500; Invitrogen, Eugene, USA) and anti-nc82 mouse 1:10 (1:10; Developmental Studies Hybridoma Bank, lowa City, USA) overnight at 4 °C in dark. Samples were washed 30 min for 6 times with PBST and they were incubated with secondary antibodies diluted in blocking solution: Alexa 488 goat anti-rabbit (1:100; Invitrogen, Eugene, USA) and Cy3 donkey anti-mouse (1:100; Jackson ImmunoResearch Laboratories, Ely, UK). Finally, after several washes with PBST0.2%, samples were mounted using Vectashield mounting medium (Vector Laboratories, Burlingame, USA). Immunostainings were performed in both males and females with no obvious expression differences between the sexes.

Images of antennal cryosections and brain dissections were collected on a Leica TCS-SP8X Confocal Laser Microscope (Leica Microsystems) and analysed with free ImageJ software (https://imagej.nih.gov/ij/)^40^. Six brains were used to count neuron cell bodies on the *Mz317-Gal4/repo-Gal80;UAS-mCD8::GFP* genotype.

### RNA extraction and RT-PCR

Reverse transcription (RT) experiments were carried out on different developemental stages and adult tissues. Total RNA from ten embryos, one third instar larva, two 20–30 h APF pupas, 50 third antennal segments, five brains and 50 eyes, from the *wildtype* genotype (*Canton-S*), *Mz317-Gal4* and *Mz97-Gal4,UAS-Stinger* lines were isolated with the RNeasy Mini Kit (Qiagen, Hilden, Germany) according to the manufacturer's instructions. cDNA was synthesised from the isolated RNA using SuperScript™ First Strand Synthesis System Kit for RT-PCR (Invitrogen, Carlsbad, USA) with oligo dTs following the manufacturer´s instructions. Total RNA from 10 embryos, 10 larvae and 10 pupae at different time points and 3 pupae at 24 h, 48 h and 72 h APF from *Canton-S* were isolated for performing RT-PCRs in Fig. S4. The exact numbers of RT-PCR experiments are given in Table S1.

### Polymerase chain reaction (PCR) and gel electrophoresis

All primers used for the PCRs were designed using *Primer 3 Plus* software specifically for amplyfing different size fragments for cDNA and gDNA (Table 4). Primers for the four transcripts of the *Ntan1* gene were also designed specifically in the 5'UTR and 3'UTR regions of each transcript, being the transcripts: *Ntan1-RA*, *Ntan1-RB*, *Ntan1-RC* and *Ntan1-RD*, which were distinguished by size, two by two (Table 4).

**Table 4:** Primers designed for the RT-PCR gene expression analysis of *G6PD*, *Ntan1* and *Ntan1* transcripts.

Gene/transcript	Primers (5′→ 3′)	Amplicon size (bp)
*G6PD*	F: AGTCGCCTACAATGGTCTGC R:GTTCGAATCGTTGCTAACGG	gDNA: 1106 cDNA: 959
*Ntan1*	F: GGTGTGCTGCTGCAGGATGACT R: CGTAGTTAAAGGGTCCAATGC	gDNA: 901 cDNA: 685
*Ntan1-RA*	F: GTGCACGAATCGCTGAACT R: CGTTCCCAGTTCAGTTCAGATCCAT	cDNA:1136
*Ntan1-RB*	F: GTCTCTCTTTCCGGTTTGCGTC R: CGTTCCCAGTTCAGTTCAGATCCAT	cDNA: 1336
*Ntan1-RC*	F: GTGCACGAATCGCTGAACT R: CGTTCCCAGTTCAGTTCAGATCCAT	cDNA: 1405
*Ntan1-RD*	F: GTCTCTCTTTCCGGTTTGCGTC R: CGTTCCCAGTTCAGTTCAGATCCAT	cDNA: 1601

The corresponding amplicon size for each primer pair is also indicated.

PCR was carried out in a final volume of 20 µl using 2 µl of template cDNA and Taq polymerase (Promega, Madison, USA) according to the manufacturer's recommendations. Samples were subjected to 35 cycles of PCR. Each cycle included 30 s of denaturation at 95 °C, 45 s of banding at 58 °C and 30 s of elongation at 72 °C for the *Ntan1* gene and 90 s of elongation at 72 °C for the transcripts. After amplification, 20 μl of the amplification products were analysed by gel electrophoresis on a 2% agarose gel with a DNA ladder according to the amplified DNA size of the sample (Table 4).

### Splinkerette method

The insertion site of the Gal4 element of the *Mz317-Gal4* line was inferred using the Splinkerette PCR method previously described^17^ with slight modifications. In short, a standard phenol–chloroform genomic DNA extraction was performed using 20 adult flies from each genotype: *Mz317-Gal4* and *GH146-Gal4* (used as a positive control). Purified DNA was digested with PsuI (BstYI) enzyme (Thermo Scientific, Lithuania, EU) at 37 °C overnight, and then inactivated for 20 min at 80 °C. The digested DNA was ligated (T4 DNA Ligase, New England BioLabs, Ipswich, USA) to double stranded splinkerette oligonucleotide^17^ for 2 h at room temperature in a total volume of 50 µl. Then, ligated DNA was used as a template to amplify both the 3′ and 5′ P-element insertion sites using primers 3′SPLNK#1 (CACTCAGACTCAATACGACAC) and 3′SPLNK#2 (GGATGTCTCTTGCCGAC) and primers 5′SPLNK#1-GAWB (TGGGAGAGTAGCGACACTCC) and 5′SPLNK#2-GAWB (GAGCTTTTTAAGTCGGCAAATATCG) respectively. After amplification, 20 μl of the amplification products were analysed by gel electrophoresis on a 2% agarose gel with a DNA ladder according to the amplified DNA size of the sample (Fig. S1). The gel band obtained from the 3′ P-element insertion site of *Mz317-Gal4* was extracted, purified and Sanger sequenced using the 3′SPLNK-SEQ (CGGGACCACCTTATG) primer by the Servicios Científico-Técnicos from the University of Oviedo using standard methods (Fig. S2). The sequence generated is available in the NCBI repository (GenBank OP158015).

## Supplementary Information

Supplementary Information.

## Data Availability

All data supporting the results in this work are available from the corresponding author on request. The Mz317-Gal4 3′ insertion site sequence generated is available in the NCBI repository with the accession number GenBank OP158015 (https://www.ncbi.nlm.nih.gov/genbank/). Not-cropped original images of all the gel electrophoresis are provided in the Supplemental Data.
