# Supplementary material for: The Ntan1 gene is expressed in perineural glia and neurons of adult Drosophila
Source: Sci Rep. 2022 Aug 30;12:14749. doi: 10.1038/s41598-022-18999-8 (PMC9427837; doi:10.1038/s41598-022-18999-8)
Supplement: Supplementary file 1 — Supplementary Information. [file 41598_2022_18999_MOESM1_ESM.pdf]

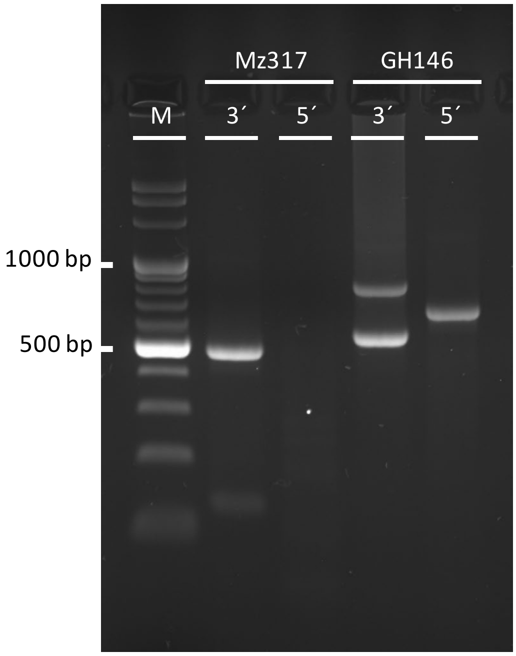

Figure S1. Representative agarose gel of PCR products of Splinkerette PCR reactions. Genomic DNA was digested with PsuI (BstYI) and subjected to splinkerette PCR to amplify the 5´ or 3´ flanking genomic DNA of the P-element insertion site. M: Molecular marker. Mz317: Genomic DNA amplicon from the *Mz317-Gal4* genotype, GH146: Genomic DNA amplicon from the *GH146-Gal4* genotype. The band obtained for Mz317 was extracted from the agarose gel, purified and DNA sequenced by regular Sanger sequencing**.**

Supplementary Figure 2

>3´Mz317-Gal4

TACCGGCAACAAGCATGCGCTCCCTTTCTGCCCAACGCACGCTCAACTGAAAACTGTTCCAAATGCTTTG
GCGCTGGCGCTTCTCTCTTCTATTCTCGCCGAGTGTCTTTTGGGCCCTCCATTCGCCAGTCGACTTCGAC
TTGGACTTTCGGCTCAGTTGCTTTTCAGTCTCTTTCCGGTTTGCGTCTCGTCGTCTCGCGGAAACTTTGG
CGCACACGCAGTTCCAGACAAATACAATTTAGAATTAAATAATATTTAAAAACACAAGTGTTACTAAAAG
TTTCCTAAAAAACGATTGTTAAGAAATGCCGATGGCTTTGTGAAAATCAGTGAAGATAACAATCCTTAAG
CTAAAGTCTATATACATAGATCCCACTAGTGTCGACACCAGTCTCA

Figure S2. Sequencing results of the 3’ P-element insertion site of *Mz317-Gal4* line.

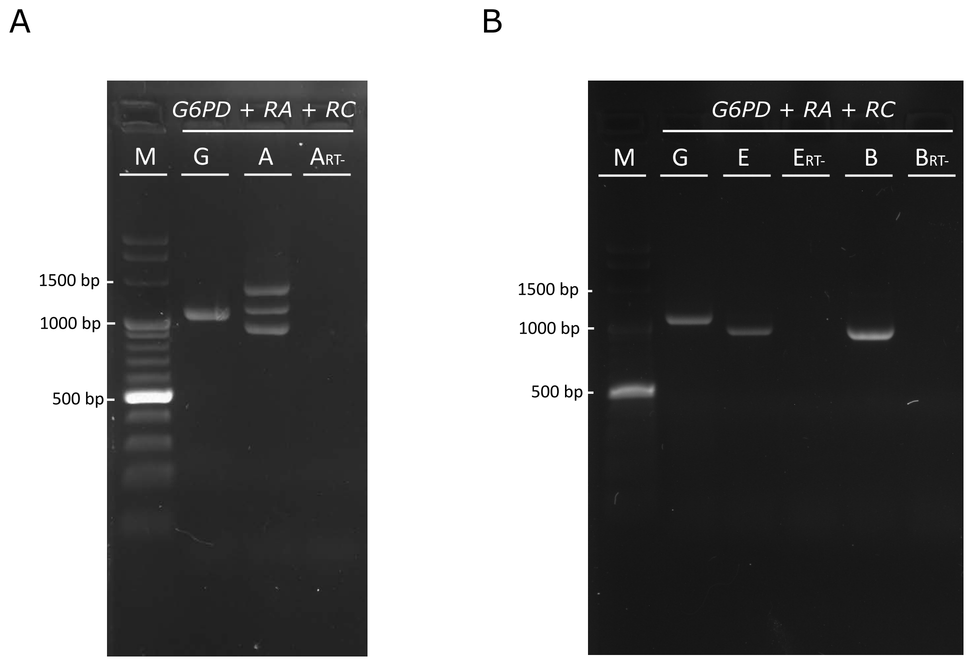

Figure S3. Gene expression analysis of *G6PD* and *Ntan1* transcripts in *Mz97-Gal4* fly line. (A) Expression of the *G6PD* housekeeping gene and *Ntan1-RA* and *Ntan1-RC* transcripts by RT-PCR in third antennal segments of *Mz97-Gal4* fly line. (B) Expression of the *G6PD* housekeeping gene and *Ntan1-RA* and *Ntan1-RC* transcripts by RT-PCR in embryos and brains of *Mz97-Gal4* fly line. M: Molecular Marker; G: Genomic DNA; A: Third Antennal Segments cDNA; E: Embryo cDNA; B: Brain cDNA; RT-: RT-PCR without retrotranscriptase.

A B

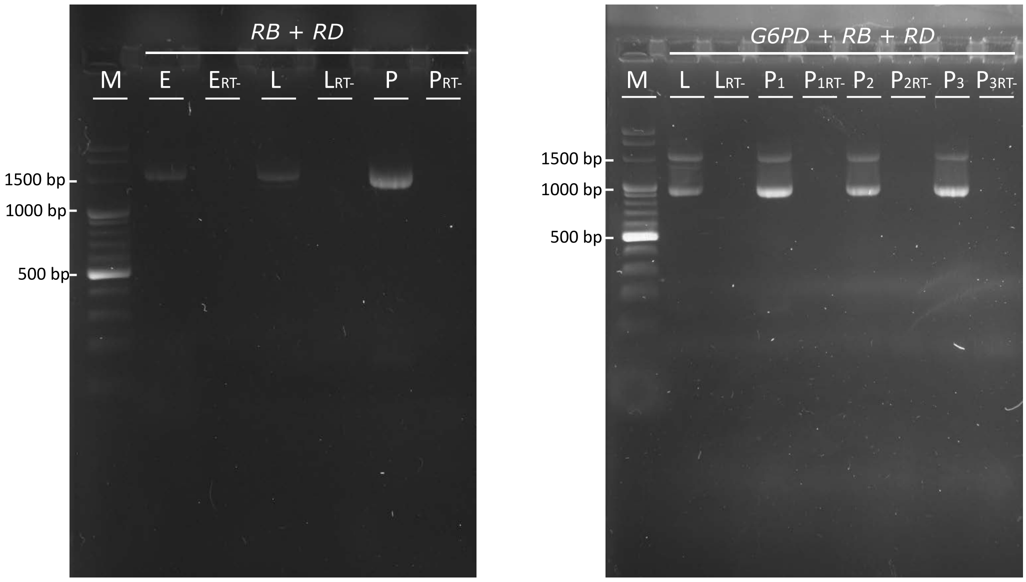

Figure S4. Gene expression analysis of *Ntan1* transcripts in *wildtype* fly line. (A) Expression of *Ntan1-RB* and *Ntan1-RD* transcripts by RT-PCR of a mixture of embryos, larvae and pupae at different time points in *wildtype* (*Canton-S*) fly line. (B) Expression of the *G6PD* housekeeping gene and *Ntan1-RB* and *Ntan1-RD* transcripts by RT-PCR in larvae and pupae at different time points in *wildtype* (*Canton-S*) fly line. M: Molecular Marker; E: Embryo cDNA; L: Larva cDNA; P_1: 24h APF cDNA; P_2: 48h APF cDNA; P_3: 72h APF cDNA; P: Pupa cDNA; RT-: RT-PCR without retrotranscriptase.

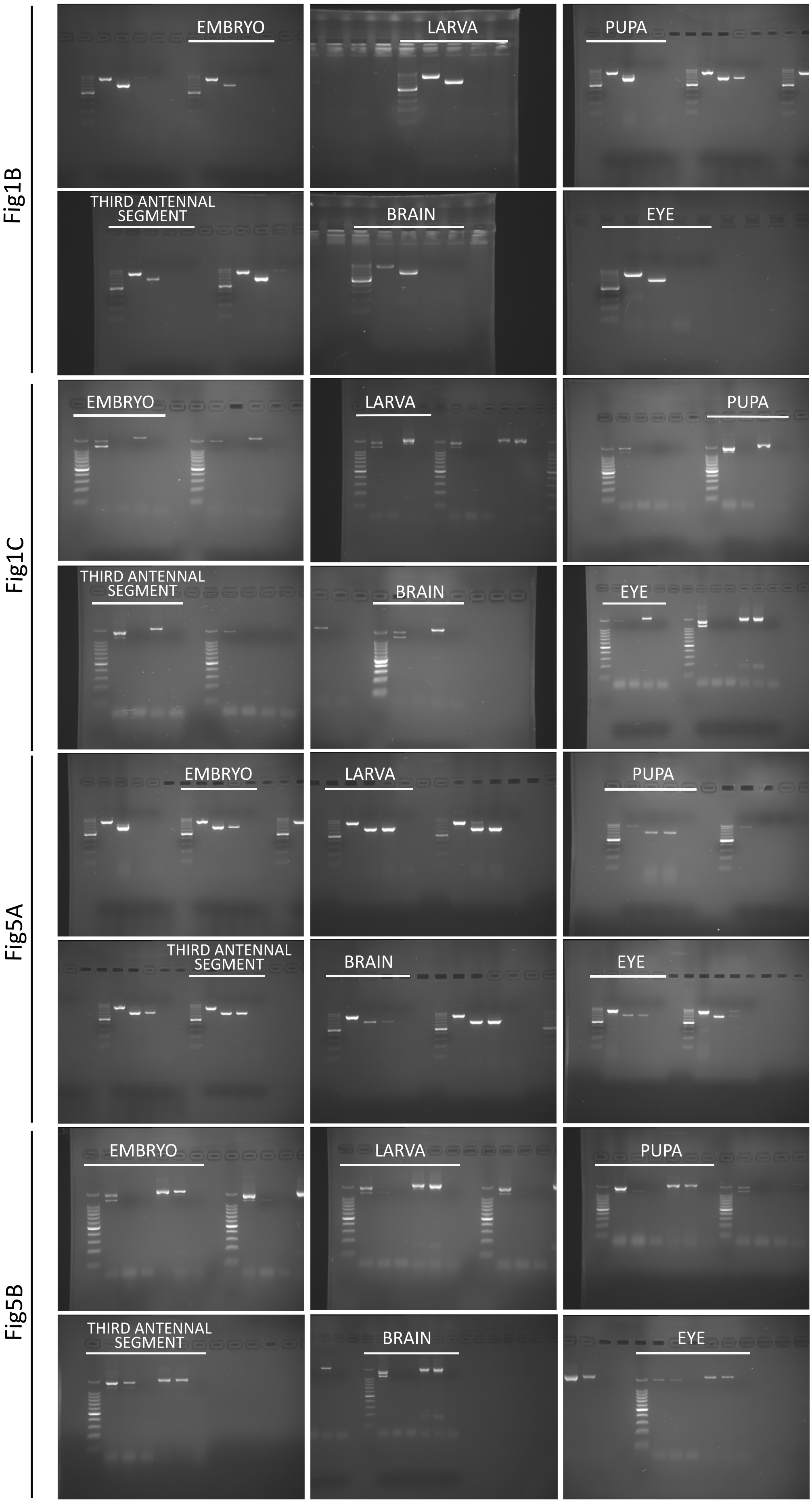

Figure S5. Original not-cropped images of the gel electrophoresis in Figures 1 and 5. The selected area of each image is under a white line. The rest of the lanes in the images are irrelevant.

Supplementary Figure 6

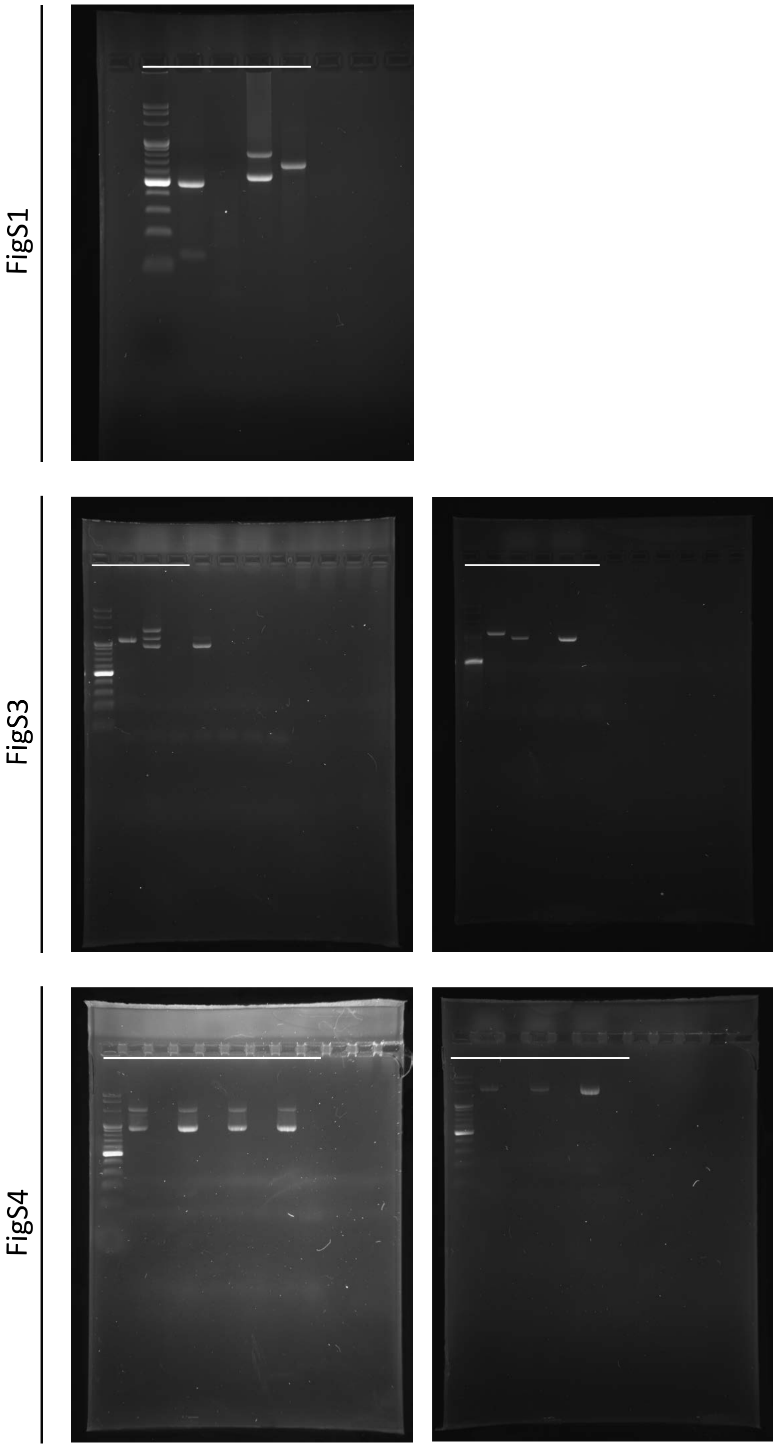

Figure S6. Original not-cropped images of the gel electrophoresis in Supplementary Figures 1, 3 and 4. The selected area of each image is under a white line. The rest of the lanes in the images are irrelevant.

Table S1. Summary of the number of repetitions for each RT-PCR for the *Ntan1* gene and its transcripts in the different fly lines (*wt*, *Mz317-Gal4* and *Mz97-Gal4*) for each developmental stage and organ studied. *No expression of one or more transcripts.

	GENE		TRANSCRIPTS	
	wt	***Mz317 and Mz97***	***wt***	***Mz317 and Mz97***
EMBRYO	1	1	2*	3*
LARVA	3	1	4	4*
PUPA	2	2	4*	4*
BRAIN	4	1	6	2*
EYE	2	1	3*	1*
THIRD ANTENNAL SEGMENT	3	1	4*	3*
